# Plasma exchange for two patients with autoimmune GFAP astrocytopathy with rapid progression to respiratory failure: a case report

**DOI:** 10.3389/fimmu.2023.1265609

**Published:** 2023-10-05

**Authors:** Jing Du, Shugang Cao, Lan Xia, Qi Li, Yanghua Tian

**Affiliations:** ^1^ Department of Neurology, Second Affiliated Hospital of Anhui Medical University, Hefei, China; ^2^ Department of Neurology, The Second People’s Hospital of Hefei, Affiliated Hefei Hospital of Anhui Medical University, Hefei, China

**Keywords:** glial fibrillary acidic protein astrocytopathy, respiratory failure, plasma exchange, intravenous methylprednisolone, intravenous immunoglobulin, efficacy

## Abstract

**Background:**

Patients with autoimmune glial fibrillary acidic protein (GFAP) astrocytopathy can present with early neurological deterioration, but rapidly progressive respiratory failure is rarely reported. We present the cases of two patients with autoimmune GFAP astrocytopathy who experienced rapid progression to respiratory failure and were effectively treated using plasma exchange therapy.

**Case report:**

Two patients were diagnosed with autoimmune GFAP astrocytopathy. Their initial symptoms were consistent with those of previously observed cases of autoimmune GFAP astrocytopathy. However, they experienced rapid progression to respiratory failure due to their lesion location. Specifically, case 1 had lesions in the medulla oblongata, and case 2 had lesions in the high cervical spinal cord, which are both common sites of lesions causing respiratory failure. The patients did not respond well to intravenous methylprednisolone and intravenous immunoglobulin initially and could not be withdrawn from ventilator support. Fortunately, subsequent plasma exchange therapy led to significant clinical improvements and successful withdrawal from ventilator support.

**Discussion:**

Patients with autoimmune GFAP astrocytopathy can present with rapidly progressive respiratory failure. Early treatment with plasma exchange can be beneficial in withdrawing patients from ventilator support.

## Introduction

Autoimmune glial fibrillary acidic protein (GFAP) astrocytopathy is a recently identified autoimmune disease of the central nervous system (1). The presence of GFAP-IgG is considered a specific biomarker of this disease. The predominant clinical manifestation of autoimmune GFAP astrocytopathy involves inflammation involving the meninges, brain parenchyma or spinal cord, or a combination of these, usually with fever, headache, impaired consciousness, and positive meningeal irritation as the first symptoms (1, 2).

In a large-sample study of 102 patients with autoimmune GFAP astrocytopathy, encephalitis (42%) and meningoencephalitis (12.5%) were the most prominent clinical presentations, followed by myelitis (10.5%) and encephalomyelitis (8%) and less commonly meningitis (5%) and meningoencephalomyelitis (3%) (2). In cases of spinal cord involvement, milder sensorimotor deficits were typically observed (2, 3). However, studies conducted in China have reported significantly different clinical features of autoimmune GFAP astrocytopathy. Specifically, it mainly manifests as myelitis (68.4%) and optic neuritis (63.2%), with longitudinal extensive transverse myelitis (LETM) being particularly common (4). These differences in clinical features between Chinese and other populations merit further investigation. However, patients presenting with rapid progression to respiratory failure are rare among cases of autoimmune GFAP astrocytopathy.

A common first-line treatment for autoimmune GFAP astrocytopathy is the use of intravenous methylprednisolone (IVMP) and/or intravenous immunoglobulin (IVIg). If this initial treatment is not effective, a combination of immunosuppressive agents or rituximab (RTX) may be needed (5–7). In patients with severe cases, plasma exchange therapy can be employed to improve the prognosis (5, 6). The majority of patients (approximately 70-80%) respond well to glucocorticoid therapy and have a good prognosis, while a subset of patients may not respond to these treatments, leaving them with functional impairment or even leading to death (5–8). Herein, we present the cases of two patients with autoimmune GFAP astrocytopathy who experienced rapid progression to respiratory failure and were effectively treated using plasma exchange.

## Case presentation

Case 1 A 19-year-old male patient was admitted to our hospital due to a fever for 6 days and reduced consciousness for half a day. The patient had developed a fever 6 days prior to admission, with a maximum body temperature of 39.2 °C and poor appetite. He had received antibiotics and symptomatic treatment at another hospital, and his peak body temperature had decreased; however, his body temperature remained abnormal at admission to our hospital. Half a day prior to admission, he developed confusion, nonsensical speech, hiccups, diarrhea, and difficulty urinating. His past medical history was unremarkable. Neurological examination upon admission suggested confusion, nonsensical speech, and autonomous limb movement. The patient exhibited meningeal irritation signs upon examination, including neck stiffness, positive Kernig’s and Brudzinski’s signs, and two transverse fingers on the chin-chest test. No abnormalities were found in the tests for infection, immune indicators, anti-nuclear antibodies, anti-neutrophil cytoplasmic antibodies, or tumor markers. The blood tests for infections indicated high levels of cytomegalovirus IgG at 431.52 AU/ml (normal range < 14 AU/ml), but other viral indicators were within normal ranges.

The cerebrospinal fluid (CSF) examination revealed an elevated CSF pressure of 330 mmH_2_O and an increased white blood cell count of 108 × 10^^6^/L (normal range: 0-8× 10^^6/^L), with 90.7% monocytes and 9.3% multinucleated cells. The CSF protein level was elevated at 1100 mg/L (normal range: 150-450 mg/L), and the glucose level was 7.72 mmol/L (normal range: 2.2-3.9 mmol/L). The CSF acid-fast and ink-staining test results were negative. CSF cytology showed increased lymphocytes, with visible monocytes and a small number of neutrophils, indicating a mixed cell-type reaction, but no abnormal cells were observed. The CSF testing for infections was negative. Initial head and cervical spine MRI revealed no anomalies. He was diagnosed with viral meningoencephalitis and treated with intravenous ganciclovir (250 mg, twice a day), but his symptoms did not improve. On the fourth day after admission, his condition worsened with a coma (Glasgow Coma Scale score of 6), respiratory distress, weakened limb muscles (Medical Research Council (MRC) score: Grade 1), and difficulty in urinating and defecating. He was intubated with a ventilator for assisted breathing. Another lumbar puncture was performed, the results of which indicated high cerebrospinal fluid pressure (330 mmH_2_O) and an elevated white blood cell count (166 × 10^^6^/L), with 89.8% being monocytes. CSF protein and chlorine levels were 1122 mg/L and 114 mmol/L (normal range: 120-132 mmol/L), respectively. Acid-fast and ink-staining test results remained negative. Next-generation sequencing (NGS) tests showed the presence of Epstein-Barr virus and herpesvirus type 1. As autoimmune-related encephalitis could not be ruled out, he was treated with IVMP (500 mg/day for 5 days, 240 mg/day for 5 days, 120mg/day for 5 days) followed by sequential oral prednisolone (60 mg/day) and IVIg (25 g/day for 5 days). However, his condition did not improve significantly.

**Figure 1:**
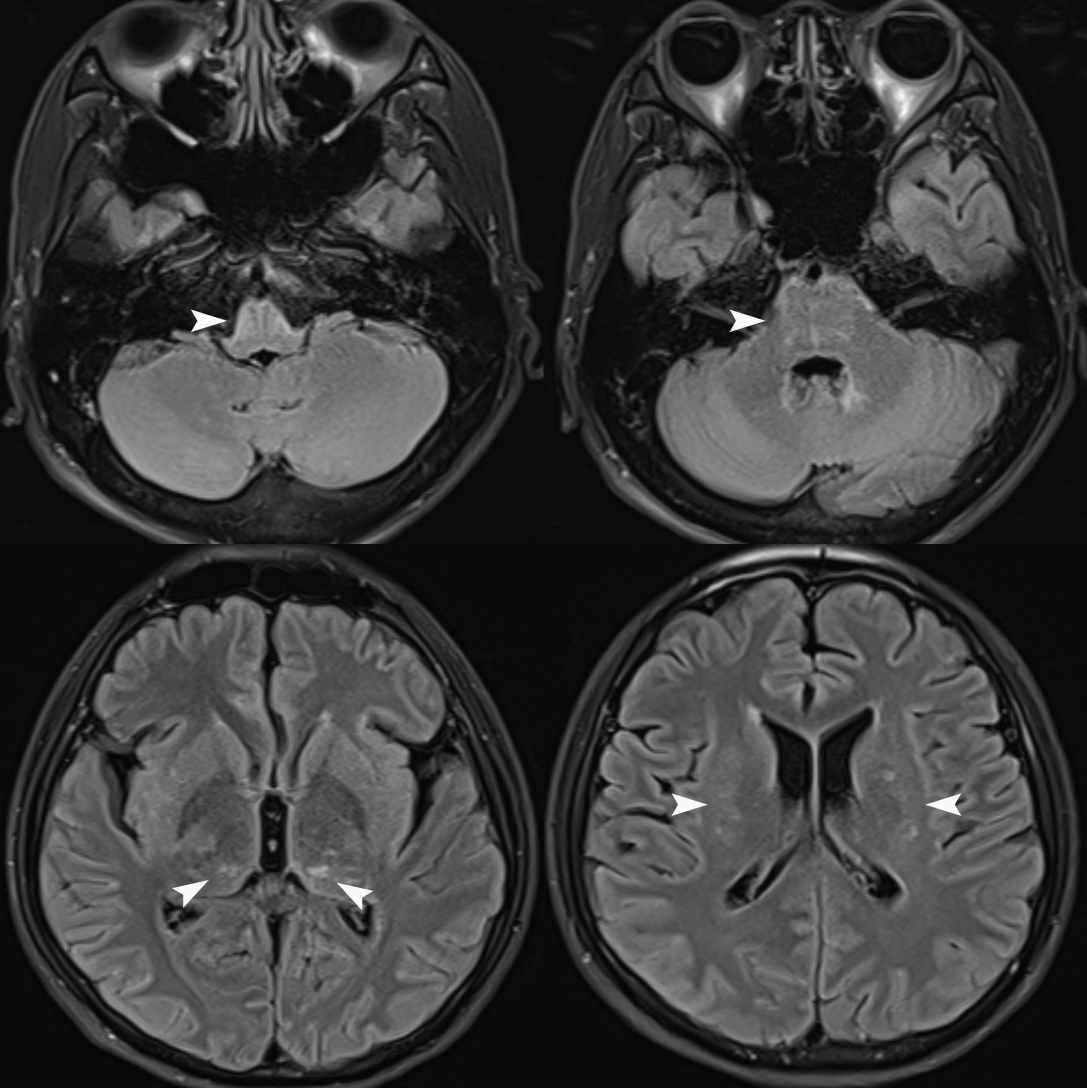
Brain MRI Flair sequence revealed abnormally high signals in the bilateral thalamus, periventricular white matter, pons, medulla oblongata, and periependymal surfaces of the fourth ventricle (white arrows) without enhancement (unshown).

We repeated the lumbar puncture and conducted a central demyelinating antibody spectrum examination, the results of which revealed high levels of GFAP-IgG in CSF (1:32, cell-based assay), while serum GFAP-IgG was negative. The results of both serum and CSF tests for aquaporin-4 IgG (AQP4-IgG), myelin oligodendrocyte glycoprotein IgG (MOG-IgG), myelin basic protein IgG (MBP-IgG), Flotillin-1/2 IgG and autoimmune encephalitis-related antibodies were negative. Additionally, the results of both serum and CSF oligoclonal band (OB) tests were negative. Serum IgG was 32.3 mg/L (normal range: 7.51-15.6 mg/L), while CSF IgG was 56.8 mg/L (normal range: 4.8-58.6 mg/L), and the IgG index was 0.36 (normal range: ≤ 0.85). Brain MRI reexamination revealed abnormally high signals in the bilateral thalamus, periventricular white matter, pons, medulla oblongata, and periependymal surfaces of the fourth ventricle without enhancement (**Figure 1**). However, no abnormal signals were observed in the whole spinal cord MRI. The patient was diagnosed with autoimmune GFAP astrocytopathy and received five plasma exchanges over 9 days (every other day). After treatment, his symptoms gradually improved, and he was successfully weaned off the ventilator. At discharge, he had regained grade 5 muscle strength in the upper limbs and grade 3 in both lower limbs. The results of the follow-up CSF test were negative for GFAP-IgG, and the results of tests for other antibodies remained negative. After discharge, he received sequential RTX (600 mg IV once every six months) to prevent relapse and then discontinued prednisolone six months later. At follow-up visits to date, his condition has largely returned to normal, except for occasional urinary urgency and he has not experienced any further relapses.

**Figure 2:**
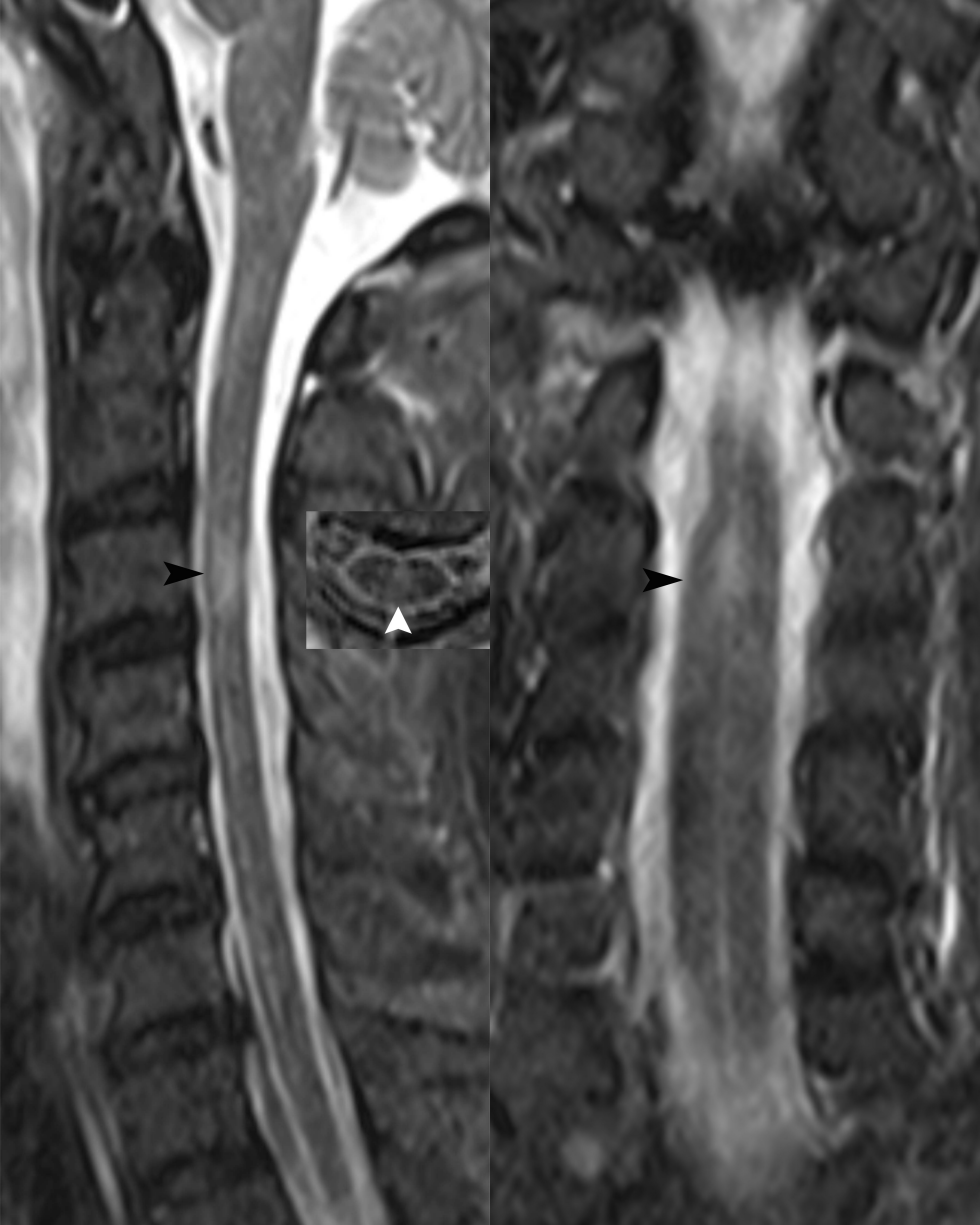
Sagittal, transverse and coronal spinal cord MRI revealed abnormal high signals in the spinal cord at the C3 level (black and white arrows).

Case 2 A 50-year-old male patient was admitted to our hospital due to fever and headache for 6 days and weakness in both lower limbs with difficulty urinating for 4 days. The patient had a fever of up to 40 °C with chills and headaches following a cold six days prior to admission. His past medical history was unremarkable. Neurological examination upon admission suggested weakened limb muscles (MRC Grade 4 in both upper limbs and Grade 0 in both lower limbs) and negative Babinski’s signs. The patient exhibited meningeal irritation signs with two transverse fingers on the chin-chest test. The test results showed no signs of any abnormalities for infections, immune indicators, anti-nuclear antibodies, anti-neutrophil cytoplasmic antibodies, thyroid function, and tumor markers. The blood tests for infections indicated high levels of rubella virus IgG at 54.16 AU/ml (normal range < 10 AU/ml), cytomegalovirus IgG at 925.72 AU/ml, and herpes simplex virus IgG at 210.8 AU/ml (normal range < 19 AU/ml), but other viral indicators were within normal ranges. CSF evaluation revealed an elevated CSF pressure of 255 mmH_2_O and an increased white blood cell count of 62 × 10^^6^/L, with 87.1% monocytes and 12.9% multinucleated cells. The CSF protein level was elevated at 1500 mg/L, the chlorine level was 109.4 mmol/L, and the glucose level was 2.99 mmol/L. The results of CSF acid-fast and ink staining tests were normal. The CSF testing for infections indicated cytomegalovirus IgG at 14.12 AU/ml. He was initially given anti-infective and antiviral treatment, IVMP (1000 mg/day), and symptomatic treatment. However, he developed respiratory failure on the fourth day after admission and was intubated with a ventilator for assisted breathing. Brain MRI showed no abnormalities, while spinal MRI revealed abnormal signals in the spinal cord at the C3 level (**Figure 2**). Unfortunately, the patient did not undergo an enhanced MRI. He was initially diagnosed with acute disseminated encephalomyelitis. We performed another lumbar puncture to complete a central demyelination antibody profile and found high levels of GFAP-IgG in the CSF (1:32, cell-based assay) and negative results for serum GFAP-IgG. The results of serum and CSF tests for other demyelinating antibodies (including AQP4-IgG, MOG-IgG and MBP-IgG) and OBs were negative. The patient was eventually diagnosed with autoimmune GFAP astrocytopathy. We treated the patient with IVMP (1000 mg/day for 3 days, 500 mg/day for 3 days, 240 mg/day for 3 days, 120mg/day for 3 days, and then sequential oral prednisolone 60 mg/day) and IVIg 37.5 g/day for 5 days, which improved his condition. However, he still required ventilation support. To address this, we performed 5 plasma exchanges over 14 days (every two days), which ultimately allowed for successful weaning off the ventilator. At discharge, his upper limb muscle strength had improved (MRC Grade 3 on the left and Grade 1 on the right), but that of both lower limbs remained at grade 0. He continues to be treated with prednisone (10 mg/day) to prevent relapse while undergoing rehabilitation. After 10 months of rehabilitation to date, his upper limb muscle strength has returned to normal (MRC Grade 5 bilaterally), but he still has poor muscle strength in both lower limbs (MRC Grade 2). Moreover, he has not yet experienced any further relapses.

## Discussion

Autoimmune GFAP astrocytopathy primarily affects the meninges, brain parenchyma, spinal cord, and optic nerve, and patients usually present with symptoms such as fever, headache, impaired consciousness, numbness and paralysis of the limbs, urinary and fecal disorders or visual loss (2). Rapid progression to respiratory failure is a rare occurrence in patients with autoimmune GFAP astrocytopathy. The initial symptoms in both patients are consistent with those of previously reported cases of autoimmune GFAP astrocytopathy. However, the rapid progression to respiratory failure can be attributed to the location of the lesions, with the lesion of case 1 involving the medulla oblongata and that of case 2 involving the high cervical spinal cord, which are both common sites of lesions causing respiratory failure. Therefore, it is crucial to take note of the lesion location in autoimmune GFAP astrocytopathy patients, as this can help anticipate the likelihood of respiratory failure and prompt the implementation of preventive and curative measures.

In imaging, autoimmune GFAP astrocytopathy typically manifests characteristic abnormally high T_2_WI and Flair signals in a radial or speckled pattern within the brainstem, spinal cord, and periventricular white matter. Enhanced MRI may reveal linear periventricular radiolucencies and/or long segmental spinal cord involvement with central enhanced lesions, but enhanced imaging results may be negative in some patients (2, 9). Additionally, in the early stages of autoimmune GFAP astrocytopathy, imaging may not show significant abnormalities (10), as was the case with the first patient where the lesion was not detected until the second examination. Therefore, a dynamic review should be considered when necessary to aid in diagnosis and management. Of note, involvement of the area postrema has been well described in neuromyelitis optica spectrum disorder (NMOSD). The presence of a lesion in the area postrema may also be another imaging hallmark of autoimmune GFAP astrocytopathy. A recent study reported eight cases of autoimmune GFAP astrocytopathy presenting with area postrema syndrome (APS) and suggested that APS could be an early but not isolated clinical manifestation of autoimmune GFAP astrocytopathy (11). However, case 1 exhibited a rare presentation of symmetrical involvement of the ventral medulla oblongata and pons, which is not commonly seen in cases of autoimmune GFAP astrocytopathy.

During the acute phase of autoimmune GFAP astrocytopathy, IVMP and/or IVIg are commonly used. However, some patients still respond poorly to these treatments and show progressive deterioration or even die (5–8). Previous studies suggest that for patients who have failed to respond to the treatments mentioned above, plasma exchange may be a beneficial alternative (5, 6). Plasma exchange uses extracorporeal blood purification techniques to separate and remove harmful substances from the blood and regulate the immune status of the body (12). For autoimmune GFAP astrocytopathy, plasma exchange may prevent the recruitment of inflammatory cells by removing cytokines, chemokines, and other inflammatory mediators from plasma and regulating the cellular immune system, which in turn attenuates the immune attack on the nervous system (5, 12, 13). A previous study had reported that a female patient who did not respond to IVMP and IVIg showed improvement after receiving plasma exchange therapy (6). In autoimmune GFAP astrocytopathy patients with high titers of CSF GFAP-IgG or extensive parenchymal or spinal cord lesions, IVMP or IVIg alone was less effective than plasma exchange therapy (5). This suggests that early implementation of plasma exchange may be more beneficial in treating these critically ill patients (5, 6). As in the present study, two patients did not respond well to IVMP and IVIg initially and could not be withdrawn from ventilators easily. However, subsequent plasma exchange therapy led to significant clinical improvements and successful withdrawal from the ventilator. Therefore, for autoimmune GFAP astrocytopathy patients with medullary and high cervical lesions that could lead to respiratory failure, plasma exchange can serve as a potential primary treatment and may also help in preventing respiratory failure in such patients.

In conclusion, patients with autoimmune GFAP astrocytopathy may experience early neurological deterioration such as rapidly progressive respiratory failure, which depends largely on the lesion location. Early treatment with plasma exchange can be beneficial in withdrawing patients from ventilator support and even potentially preventing respiratory failure.

## Data availability statement

The original contributions presented in the study are publicly available. This data can be found here: https://www.ncbi.nlm.nih.gov/bioproject/PRJNA1011036/.

## Ethics statement

The studies involving humans were approved by Second Affiliated Hospital of Anhui Medical University. The studies were conducted in accordance with the local legislation and institutional requirements. The participants provided their written informed consent to participate in this study. Written informed consent was obtained from the individual(s) for the publication of any potentially identifiable images or data included in this article.

## Author contributions

JD: Conceptualization, Investigation, Methodology, Writing – original draft, Writing – review & editing. SC: Conceptualization, Investigation, Methodology, Writing – original draft, Writing – review & editing. LX: Conceptualization, Investigation, Methodology, Writing – review & editing. QL: Methodology, Supervision, Validation, Writing – review & editing. YT: Methodology, Supervision, Validation, Writing – review & editing.
