# Corrigendum: Plasma exchange for two patients with autoimmune GFAP astrocytopathy with rapid progression to respiratory failure: a case report

**DOI:** 10.3389/fimmu.2024.1366725

**Published:** 2024-01-16

**Authors:** Jing Du, Shugang Cao, Lan Xia, Qi Li, Yanghua Tian

**Affiliations:** ^1^ Department of Neurology, Second Affiliated Hospital of Anhui Medical University, Hefei, China; ^2^ Department of Neurology, The Second People’s Hospital of Hefei, Affiliated Hefei Hospital of Anhui Medical University, Hefei, China

**Keywords:** glial fibrillary acidic protein astrocytopathy, respiratory failure, plasma exchange, intravenous methylprednisolone, intravenous immunoglobulin, efficacy

In the published article, there was an error in affiliation of the author Qi Li. Instead of “Qi Li^2^, it should be Qi Li^1^”.

The authors apologize for this error and state that this does not change the scientific conclusions of the article in any way. The original article has been updated.

